# Effects of Peer and Teacher Support on Students’ Creative Thinking: Emotional Intelligence as a Mediator and Emotion Regulation Strategy as a Moderator

**DOI:** 10.3390/jintelligence13050053

**Published:** 2025-04-25

**Authors:** Yafei Shi, Qi Cheng, Yantao Wei, Yunzhen Liang, Ke Zhu

**Affiliations:** 1 Faculty of Education, Henan Normal University, Xinxiang 453007, China; yafeishi@mails.ccnu.edu.cn (Y.S.);; 2 Henan Collaborative Innovation Center for Intelligent Education, Henan Normal University, Xinxiang 453007, China; 3 School of Physics, Henan Normal University, Xinxiang 453007, China; 4 Faculty of Artificial Intelligence in Education, Central China Normal University, Wuhan 430079, China

**Keywords:** peer support, teacher support, creative thinking, emotional intelligence, emotion regulation strategies

## Abstract

This study aimed to explore the relationships among peer and teacher support, emotional intelligence, and creative thinking. A total of 335 middle school students in grade seven were surveyed in China, including boys 187 (55.8%) and girls 148 (44.2%), aged from 11 to 14 years (M = 12.5; SD = 0.5). Results of the partial least square structural equation modeling showed that emotional intelligence was a positive mediator in the processes from peer and teacher support to middle school students’ creative thinking, and emotion regulation strategies moderated these processes from emotional intelligence to creative thinking. Specifically, both peer and teacher support had an indirect effect on creative thinking through emotional intelligence. Moreover, the four dimensions of emotional intelligence bore different mediating powers. Among them, emotion regulation exhibited the greatest mediating power, and self-emotion appraisal is the least. In addition, both reappraisal and suppression positively moderated the impact of emotional intelligence on creative thinking. Moreover, reappraisal had stronger moderating power than that of suppression. Interestingly, the direct effects of both peer and teacher support on creative thinking were not observed. This study offers knowledge about the mechanisms of peer and teacher support and students’ creative thinking, and implications for practitioners were also discussed in this study.

## 1. Introduction

Creative thinking benefits all aspects of students’ life and learning (Zirak and Ahmadian 2015a). Researchers have indicated that creative behavior and academic achievement could be predicted effectively and positively by creative thinking (Akpur 2020; Torrance 1972). Creative thinking is also viewed as an adjustable tool for successfully dealing with various unknown problems, which contributes to constructive and flexible behaviors in these novel and demanding environments (Kashani-Vahid et al. 2017). Therefore, it is essential to cultivate young learners’ creative thinking skills to prepare them for study and future work.

An increasing line of research has suggested the potent role of classroom environment, particularly teacher and peer support, in the development of students’ creative thinking (Gherasim et al. 2013; Lamb 2020; Luan et al. 2023; Zou et al. 2023). For instance, evidence from Zhang et al. (2020) indicates that perceived teacher support could contribute to primary students’ creative thinking, and creative self-efficacy mediated the relationship between teacher support and students’ creative thinking. However, how teacher and peer support influence students’ creative thinking and what is its internal mechanism still received less attention (Dietrich et al. 2015). In addition to classroom environmental factors, individual factors may also affect students’ academic performance and creative thinking. Emotional intelligence is one type of social intelligence that stresses the ability to manage, evaluate, and utilize affective information (Salovey and Mayer 1990). Research has shown that emotional intelligence is significantly associated with students’ academic performance (Chew et al. 2013; Fernandez-Perez and Martin-Rojas 2022). Moreover, the role of emotional intelligence on students’ creative thinking has been attracting more attention over the last few years (Ebrahimi et al. 2018; Giancola et al. 2022; Li et al. 2021). Evidence has revealed that there is a complicated relationship between emotional intelligence and creative thinking (Afshar and Rahimi 2016; Durnali et al. 2023; Şahin et al. 2016). However, the effect of emotional intelligence on creative thinking has tended to be ignored in teaching and learning processes (Hargreaves 2000). The relationship between social support and emotional intelligence also received attention from researchers (e.g., Hogan et al. 2010; Kong et al. 2012). However, little is known regarding mechanisms by which teacher and peer support influence students’ creative thinking through emotional intelligence. Therefore, exploring the relationships among teacher and peer support, emotional intelligence, and creative thinking is necessary.

To address the research gap mentioned above, this study explored the antecedents of creative thinking and mechanisms among them in educational contexts from the quantitative analysis perspective. The current study aims to shed light on the interaction between social support and creative thinking and contribute to some implications for practitioners to cultivate students’ creative thinking in teaching and learning processes.

## 2. Theoretical Framework and Model Development

### 2.1. Creative Thinking

Creative thinking refers to the ability to generate new ideas or solutions when solving problems (Hadar and Tirosh 2019). It is believed to be the dynamic energy behind all human progress (Ramón and Chacón-López 2021; Saggar et al. 2017). In addition, middle school students are at a golden stage for the development of creativity, thinking ability, and higher-order thinking (Gong 2020). Therefore, cultivating middle school students’ creative thinking has become essential in developing an affordable educational system (Mrayyan 2016). Different forms of creative thinking are usually mentioned, such as imaginative thinking, reverse thinking, divergent thinking, and so on (Xiong et al. 2022; Zhang et al. 2020). The creative thinking in this study is evaluated according to the six major dimensions, including innovation search, courage, self-discipline, inquisitiveness, doubt, and flexibility (Ayyildiz and Yilmaz 2021; Durnali et al. 2023; Özgenel and Çetin 2017).

Creative thinking can be affected by both individual and environmental factors (Yildiz and Yildiz 2021; Zhang et al. 2020). For instance, in terms of individual factors, Zirak and Ahmadian (2015b) suggested that fifth grade students’ creative thinking could be developed by different dimensions of individuals’ emotional intelligence. Avcı and Durak (2023) identified that undergraduate students’ extrinsic motivation positively predicted their creative thinking dispositions. In terms of environmental factors, Zhang et al. (2020) observed that perceived teacher support significantly positively predicted primary students’ creative thinking. Liu et al. (2023) considered that social support from peers could afford senior high school students’ creative behaviors. However, little research explores the mechanisms of how and why external classroom environment factors (e.g., peer and teacher support) affect middle school students’ creative thinking in educational contexts. Specifically, the present study aimed to explore the relationships among environmental factors (e.g., peer and teacher support), emotional intelligence, and creative thinking.

### 2.2. Peer Support and Creative Thinking

Peer support is regarded as a series of behaviors of giving and receiving what is helpful based on fundamental principles such as mutual respect and consensus agreement (Mead et al. 2001). Peer support plays a significant role for individuals with similar life and learning experiences to conquer complex and difficult problems (Mead and MacNeil 2006; Mishra 2020). Peer support, including social, emotional, and academic aspects, may likely facilitate students’ academic performance and health (Caporale-Berkowitz 2022; Tucker et al. 2020). Generally, peer support involves very important aspects, such as listening, building intimate relationships, giving and receiving help, and improving communication (Caporale-Berkowitz 2022). In education contexts, peer support mainly concerns social-emotional support, usually combined with instrumental support (Solomon 2004). For instance, Tucker et al. (2020) found that peer academic support could significantly affect university students’ success. Brouwer et al. (2022) found that university students with good performance tend to actively establish peer academic help and friendship relationships. Olana and Tefera (2022) revealed that peer support regarding emotional, instrumental, appraisal, and informational aspects strongly predicted secondary school students’ school engagement. Furthermore, a meta-analysis demonstrated that peer support interventions offered meaningful effect on students’ academic performance (Yan et al. 2022). Therefore, peer support could provide valuable practical, affective, and social assistance to individuals (Gidugu et al. 2015; Hogan et al. 2010).

All kinds of peer support are perceived as beneficial (Gidugu et al. 2015; Mitchell 2023; Zhong et al. 2021). For instance, peer emotional support could offer motivation, hope, and encouragement to individuals who encounter difficulties and challenges (Gidugu et al. 2015; Dawson and Samek 2022). Ruzek et al. (2016) reported that perceived supportive and respectful interaction from peers in Winter could encourage middle school students’ behavioral engagement and learning motivation in the following Spring. Kaynak et al. (2023) explored the psychological mechanism from middle school students’ perceived peer support to school achievement with the mediating role. Results concluded that students’ school motivation fully meditated the relationship between their perceived peer support and achievement, as well as that this association was moderated by gender. Peer support also plays a significant role in students’ creative thinking (Segundo-Marcos et al. 2023). For instance, Bulut (2019) explored the effect of peer instruction on creative thinking of college students with a quasi-experimental design, and found that peer instruction enhanced students’ creative thinking skill. Winks et al. (2020) indicated that campus design principles affording faculty peer learning could nurture creativity and innovation, such as designing communal spaces with social functions and collaborative spaces with collective culture. Lee et al. (2021) found that rubric based peer-feedback strategy could significantly enhance primary school students’ creative thinking and metacognitive awareness. Zhan et al. (2023) conducted a meta-analysis to investigate the effects of peer assessment on students’ high-order thinking. Results concluded that online peer assessment exerted significant contribution to students’ critical thinking, reasoning and reflective thinking. Therefore, based on the analysis above, it was proposed that peer support would be positively associated with middle school students’ creative thinking.

### 2.3. Teacher Support and Creative Thinking

Teacher support is defined as the students’ perception that teachers care about, value, and support them (Patrick et al. 2007; Tao et al. 2022). Some researchers have claimed that teacher support features a multifaceted structure, including emotional, instrumental, and informational support (Federici and Skaalvik 2014; Semmer et al. 2008; Tao et al. 2022). Students who feel that teachers care more about them will exhibit higher academic achievement and better performance (Federici and Skaalvik 2014; Shin and Chang 2022). Moreover, teacher support, whether emotional or academic, would be effectively conducive to students’ self-regulation strategies, task-related interaction, and academic performance (Hornstra et al. 2021; Kashy-Rosenbaum et al. 2018; Patrick et al. 2007). For instance, Zhang and Zou (2024) identified two models of teacher support in self-regulated L2 learning, including self-regulated L2 learning instruction and self-regulated L2 learning guidance, the effectiveness of which was examined in promoting learning efficiency, spontaneity, and proficiency. Affuso et al. (2023) concluded that secondary school students perceived that teacher support and parental monitoring could significant influence their self-determined motivation and self-efficacy, and in turn affect their academic performance. Similarly, a meta-analysis by Tao et al. (2022) observed that emotional support from teachers contributed to a larger effect on students’ achievement than that of autonomy and academic support.

Moreover, teacher support is also helpful in students’ creative thinking abilities (Giacumo and Savenye 2020; Mrayyan 2016; Soydan Oktay and Yüzer 2023). Kaur (2017) suggested that instructional strategies of De Bono’s six thinking hats could serve as effective approach for cultivating students’ creative thinking and problem-solving abilities in digital age. Orakci and Durnali (2023) explored relationships between teachers’ autonomy support, creative thinking, metacognition, and self-efficacy. Results showed that teachers’ autonomy support positively affected creative thinking and metacognition. Hu et al. (2016) found that virtual reality-integrated creative thinking instruction presented significant effect on university students’ creative thinking (e.g., sensitivity and fluency). Also, Gregory et al. (2013) reviewed the literature about cultivating creative thinking in the educational context. Results suggested that creative thinking could be developed through specific teacher support, such as offering a wealth of information, asking questions to encourage idea generation, and supporting collaboration to solve multifaceted problems. Similarly, Yuan et al. (2019) also indicated that teacher encouragement and intrinsic motivation indirectly influence high school students’ creativity with a mediating role of creative process engagement. Zhang et al. (2020) proposed that teacher support would directly facilitate primary students’ creative thinking, and this relationship was meditated by creative self-efficacy. Therefore, this study posited that middle school students’ perceived teacher support would be positively associated with their creative thinking.

### 2.4. Mediating Role of Emotional Intelligence

Emotional intelligence stresses the ability to evaluate, discriminate, and use perceived affective information (Naidu et al. 2023; Salovey and Mayer 1990). Mayer et al. (2008) also viewed emotional intelligence as integrating intelligence and emotion to regulate one’s thoughts. That is, individuals with a higher level of emotional intelligence would be seen as being more pleasant, empathetic, and sociable (Salovey and Mayer 1990). Not only does emotional intelligence process and enhance one’s emotions, but it can also promote one’s abstract thinking and problem-solving abilities (Valente et al. 2020). Generally, emotional intelligence is often seen as a multifaceted concept featuring three mental processes involving appraising and expressing individuals’ and others’ emotions, regulating individuals’ and others’ emotions, and using emotions (Fino et al. 2023; Salovey and Mayer 1990). Specifically, self-emotional appraisal concerns individuals’ ability to understand and exhibit emotions. Other’s emotional appraisal denotes people’s ability to understand and perceive others’ deep emotions. Regulation of emotion refers to the ability to regulate and manage one’s and others’ emotions, including the up-regulation of positive emotions and down-regulation of negative emotions. The use of emotion stresses one’s ability to employ affective information to guide actions involving constructive activities and promote individual performance.

Previous research revealed significant relationships between teacher and peer support and emotional intelligence (e.g., Di Fabio and Kenny 2015; Hogan et al. 2010). For instance, a study conducted by Hogan et al. (2010) in Canada found that grade 10 students’ perceived social support from peers significantly predicted their emotional intelligence, while family support did not. Fabio and Kenny (2012) demonstrated that individuals who perceived more support from peers would be better to recognize others’ emotion, express their own emotion, and use their emotions to solve problems. They found that Italian high school students’ perceived peer support and social support from friends would positively impact their emotional intelligence. Teacher support is another important factor affecting students’ emotional intelligence in the classroom environment. For instance, Dubovyk et al. (2020) raised some issues that future teachers should pay attention to in teaching to develop students’ emotional intelligence, such as embedding educational games, participating in the game with students, and setting tasks affording students’ skill in identifying and evaluating emotions. Pool and Qualter (2012) designed a teaching program to support university students’ emotional intelligence and emotional self-efficacy based on Salovey and Mayer Four Branch Model in England. Results found that university students’ emotional intelligence and emotional self-efficacy were significantly enhanced both in male and female participants. Therefore, peer and teacher support are correlated to emotional intelligence. In addition, emotional intelligence is viewed as a significant predictor of academic performance and success (Sánchez-Álvarez et al. 2020; Song et al. 2010). Researchers also suggested that individuals proficient at managing emotions commonly own the ability to use positive emotions to influence and foster their creativity (Giancola et al. 2022; Isen et al. 1987; Siu and Wong 2016). People with emotional intelligence would engage in the creative process and show more creative behavior, influencing their creativity and facilitating their creative thinking (Darvishmotevali et al. 2018; Durnali et al. 2023; Jafri 2018). Li et al. (2021) evaluated the relationship between emotional intelligence and creative thinking among 269 medical undergraduates. The findings of linear regression analysis concluded that undergraduates’ emotional intelligence significantly predicted their creative thinking. Therefore, the present study posited that middle school students’ emotional intelligence would mediate the relationship between peer and teacher support and creative thinking.

### 2.5. Moderating Role of Emotion Regulation Strategies

Emotional regulation refers to the psychological and physical processes used to manage, estimate, and modify emotional reactions (Thompson 1994). There is a growing appreciation that individuals can influence their emotions using different emotional regulation strategies (Besson et al. 2023; Dixon-Gordon et al. 2015; Gross and John 2003). In educational contexts, researchers found that students who perceived more emotional support or academic assistance from teachers or peers would exhibit a stronger tendency to employ self-regulatory strategies (Lemberger-Truelove et al. 2018; Merritt et al. 2012; Patrick et al. 2007). Generally, emotion regulation strategies can be divided into two dimensions including reappraisal and suppression (Gross and John 2003). These two emotional strategies may result in different consequences (Chacón-Cuberos et al. 2021; Gao and Yang 2023). Specifically, the former occurs earlier to alter the entire subsequent emotional tendency (Gross and John 2003), while the latter emerges during the emotion-generative process, so it commonly functions well in decreasing the behavioral expression of negative emotions (Bariola et al. 2011; Chacón-Cuberos et al. 2021; Gao and Yang 2023). Giancola et al. (2024) explored the mediating role of young adults’ emotion regulation strategies in the relationship between openness to experience and divergent thinking. Results showed that cognitive reappraisal played a significant mediating role between openness to experience and divergent thinking (fluency, flexibility, and originality), while the mediating role of emotion suppression was not observed. Therefore, emotion regulation strategies could effectively influence students’ learning processes and the teaching quality (Mendzheritskaya and Hansen 2019; Zyberaj 2022).

Existing studies have indicated that students’ emotion regulation ability is highly associated with their creativity (Ivcevic and Brackett 2015; Wu et al. 2020; Yousaf and Taylor 2022). For instance, Yeh and Li (2008) argued that emotion regulation strategies and positive temperament positively affected children’s creativity. However, although emotional intelligence could influence creative thinking, there may be differences under different conditions. For instance, Wang et al. (2024) explored the psychological mechanisms between justice sensitivity of college students and their malevolent creativity with the moderating role of emotion regulation strategies. Results indicated that both trait anger and state anger positively mediated the relationship between justice sensitivity and malevolence, and the effect of justice sensitivity on malevolent creativity could be indirectly moderated by emotion. Wang and Jiang (2022) explored the effects of negative emotion on university students’ creativity, with the mediating role of frustration tolerance and moderating role of emotion regulation strategies. Results concluded that university students’ frustration tolerance positively mediated the association between negative emotion and creativity. In addition, expressive suppression positively moderated this relationship, while cognitive reappraisal exerted a negative moderating effect. The gender difference was observed in the relationship between adults’ emotional intelligence and creativity (Zia and Rouhollahi 2020). Darvishmotevali et al. (2018) demonstrated that cultural intelligence could moderate the association between frontline employees’ emotional intelligence and creative performance. Emotion regulation strategies may also regulate the effect of emotional intelligence on creative thinking through affecting positive emotion. For instance, positive emotions increase the fluency of thinking, and negative emotions stimulate the originality of creative thinking because of the regulated effects of emotion (Wu et al. 2020). Parke et al. (2015) interpreted how and why emotional intelligence shaped creative thinking using multi-methods, and found that emotional intelligence may enable individuals to maintain and leverage positive emotion, enhancing creative thinking. Darvishmotevali et al. (2018) indicated that an individual’s emotional intelligence could promote their creative thinking by supporting individuals to understand the relationship between emotions and performance. Carmeli et al. (2014) verified the mediating role of employees’ positive emotion (e.g., vigor) on relationship between emotional intelligence and creative thinking through structural equation modeling analysis method. However, limited research regarding the relationship between emotional intelligence and middle school students’ creative thinking considered the influence of emotion regulation strategies. Therefore, this study proposed that emotion regulation strategies would moderate the relationship between emotional intelligence and middle school students’ creative thinking.

### 2.6. The Current Study

**Figure 1:**
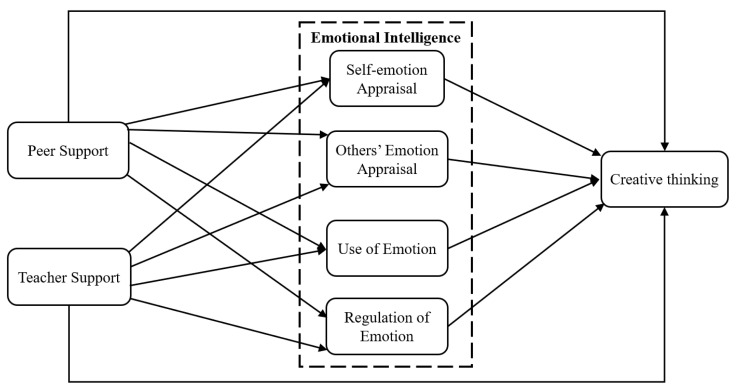
Research model in this study.

The hypotheses are as follows and the research model is illustrated in Figure 1.

**Hypothesis** **1.** *Peer support has direct effects on creative thinking (H1a), self-emotion appraisal (H1b), others’ emotion appraisal (H1c), the use of emotion (H1d), and the regulation of emotion (H1e)*.

**Hypothesis** **2.** *Teacher support has direct effects on creative thinking(H2a), self-emotion appraisal (H2b), others’ emotion appraisal (H2c), the use of emotion (H2d), and the regulation of emotion (H2e)*.

**Hypothesis** **3.** *Self-emotion appraisal (H3a), others’ emotion appraisal (H3b), the use of emotion (H3c), and the regulation of emotion (H3d) have direct effects on creative thinking*.

**Hypothesis** **4.** *Emotional intelligence mediates the association between peer support and creative thinking (H4)*.

**Hypothesis** **5.** *Emotional intelligence mediates the association between teacher support and creative thinking (H5)*.

**Hypothesis** **6.** *Emotion regulation strategies (reappraisal: H6a; suppression: H6b) positively moderate the association between emotional intelligence and creative thinking*.

## 3. Methodology

### 3.1. Participants

This study was conducted in a middle school in Zhengzhou, China, in September 2022. Permission and approval were received from school administrators and students before carrying out this survey. After explaining the research purpose, students were invited to complete a questionnaire voluntarily and anonymously. Finally, a total of 398 responses were collected. After removing invalid and missing values, 335 valid responses were obtained, and the response rate was 84.17%. The average age of participants was 12.5, with a standard deviation of 0.5. Boys and girls accounted for 55.8% (n = 187) and 44.2% (n = 148), respectively. This study was approved by the Ethical Committee of the Henan Normal University (protocol code HNSD-2023-15-06, 26 April 2023).

### 3.2. Instruments

The questionnaire employed in this study consisted of two sections. The first part collected participants’ demographic information. The other was employed to collect participants’ perceptions, including peer and teacher support, emotional intelligence, emotion regulation strategies, and creative thinking. All scales were adapted from existing, well-established measurement tools, including Torsheim et al. (2000), Wong and Law (2002), Gross and John (2003), and Özgenel and Çetin (2017). First, these scales were translated into Chinese by two doctoral students and five master students. Then, a researcher with about 15 years of research experience further adjusted it slightly to fit into the knowledge background of middle school students. Finally, the confirmatory factor analysis (CFA) was used to test these constructs more stringently.

#### 3.2.1. Teacher Support Scale

All four items of teacher support were adapted from the teacher support scale (TSS; Torsheim et al. 2000). Based on the results of CFA, four items of TSS were selected to measure students’ perceived teacher support with a five-point Likert-type scale ranging from 1 (strongly disagree) to 5 (strongly agree). An example item was “when I need extra help, I can get it from our teacher.” The Cronbach’s α of the scale was 0.867. The TSS fits the data well (χ^2^ = 1.482, χ^2^/df = 0.741, RMSEA = 0.00, CFI = 1.00, TLI = 1.00, SRMR = 0.006).

#### 3.2.2. Peer Support Scale

Three items were used to measure students’ perceived peer support by peer support scale (PSS; Torsheim et al. 2000). Students rated their perception on support from their classmates on a five-point Likert scale. The higher the score, the more peer support they receive from their learning partner. Three items were adopted to measure this construct, such as “most students are kind and helpful in my class”. The Cronbach’s α of this scale was 0.891. The PSS also fits the data well (χ^2^ = 0.00, χ^2^/df = 0, RMSEA = 0.00, CFI = 1.00, TLI = 1.00, SRMR = 0.00).

#### 3.2.3. Emotional Intelligence Scale

Emotional intelligence scale consisted of four subscales, including self-emotion appraisal with three items, others’ emotion appraisal with four items, the use of emotion with three items, and the regulation of emotion with three items. The items were adapted from the emotional intelligence scale (EIS; Wong and Law 2002). In this study, Cronbach’s α values were 0.891 for self-emotion appraisal, 0.870 for others’ emotion appraisal, 0.919 for the use of emotion, and 0.931 for the regulation of emotion. The EIS fits the data well (χ^2^ = 187.242, χ^2^/df = 3.07, RMSEA = 0.079, CFI = 0.965, TLI = 0.955, SRMR = 0.043).

#### 3.2.4. Emotion Regulation Strategies Scale

Emotion regulation strategies scale used in this study includes reappraisal and suppression factors. It was adapted from the emotion regulation questionnaire (ERQ; Gross and John 2003). For the reappraisal subscale, an example item was “When facing problems, I could control my emotions by changing the way I think about them”. For the suppression subscale, an example item was “I keep my emotions to myself”. Cronbach’s α values were 0.948 for reappraisal with five items and 0.893 for suppression with three items. The ERQ fits the data well (χ^2^ = 41.113, χ^2^/df = 2.28, RMSEA = 0.062, CFI = 0.99, TLI = 0.98, SRMR = 0.03).

#### 3.2.5. Creative Thinking Scale

The creative thinking scale in this study was adapted from Özgenel and Çetin (2017). Specifically, this scale consists of six dimensions featuring innovation search (8 items, e.g., “I produce useful and original answers or solutions to problems or situations in our class activities”), courage (4 items, e.g., “I’m not afraid of making mistakes in our class”), self-discipline (5 items, “I work disciplinedly to create an idea or product in our class”), inquisitiveness (3 items, “I am curious about what is happening around me in our class”), doubt (2 items, “I ask the “I wonder” question about a problem I encounter in our class.”), and flexibility (3 items, “I try to look at things from different perspectives in our class”). This scale was adapted to fit the context of this study, and after that, it was employed as a data collection tool to measure students’ creative thinking. Cronbach’s α values were 0.945 for innovation search, 0.856 for courage with, 0.916 for self-discipline, 0.806 for inquisitiveness, 0.758 for doubt, and 0.782 for flexibility. The creative thinking scale fits the data well (χ^2^ = 763.760, χ^2^/df = 2.84, RMSEA = 0.074, CFI = 0.930, TLI = 0.922, SRMR = 0.042).

### 3.3. Date Collection and Analysis

The data were collected by WJX (www.wjx.cn), an online questionnaire distribution tool, in September 2022. This study first obtained approval from the school administration. Subsequently, the teacher who was a member of our research team explained the purpose of the research to the students. Finally, the teacher assisted in distributing the questionnaires, following the principles of complete voluntary and anonymity. SPSS 25 and SmartPLS 3 statistics tools were employed for data analysis. The analysis consisted of three steps. First, the data were screened by excluding the invalid and careless values. Then, CFA was conducted to ensure a good indicator of reliability. Finally, the partial least square structural equation modeling (PLS-SEM) was employed to evaluate the hypothesized model.

## 4. Results

### 4.1. Assessment of Measurement Model

The reliability and validity of the measurement model were assessed using indicator loadings, composite reliability (CR), and average variance extracted (AVE). In this study, creative thinking had a second-order factor structure featuring innovation search, courage, self-discipline, inquisitiveness, doubt, and flexibility. Therefore, in order to specify the higher-order construct, the two-stage approach was employed to assess the measurement model (Sarstedt et al. 2019). First, the lower-order construct was estimated. The results of reliability and convergence validity are shown in Table 1. Most indicator loadings were greater than 0.7, and all p-values were statistically significant (*p* < 0.001), indicating that the indicator of reliability was established (Hair et al. 2012). The CR values of latent constructs ranged from 0.872 to 0.954, exceeding the recommended cut-off value of 0.7 (Bagozzi and Yi 1988). AVE values were greater than 0.6, meeting the recommended criteria of 0.5 (Fornell and Larcker 1981). Therefore, the reliability and convergence validity of the lower-order construct was established in this study. Table 2 shows that the correlation coefficients between each latent construct were less than the square roots of AVE, which indicated that the discriminant validity of lower-order constructs was adequate.

In the second stage, the estimated scores of the lower-order constructs, including innovation search, courage, self-discipline, inquisitiveness, doubt, and flexibility, were used to develop the higher-order construct. The results of the assessment for the measurement model in the second stage are presented in Table 3. All indicator loading, CR, and AVE values exceed the required threshold. Therefore, the construct reliability was adequate in this study. Table 4 illustrates the results of discriminate validity in the second stage, indicating that the discriminant validity of the measurement model was established in this study.

### 4.2. Assessment of Structural Model

The structural model was assessed using the coefficient of determination (R^2^), effect size (f^2^), predictive relevance (Q^2^), and path coefficients. The recommended cut-off thresholds are R^2^ > 0.19 (Chin 1998), f^2^ > 0.02 (Chin 1998), and Q^2^ > 0 (Hair et al. 2017). As shown in Table 5, all the values of Q^2^ were greater than 0, indicating that the structural model’s predictive relevance was acceptable. R^2^ of endogenous variables ranged from 0.311 to 0.699, which meant that endogenous latent variables could be explained well in this model. The bootstrapping method was employed to test the levels of significance of the standardized path coefficients with 5000 bootstrap iterations.

**Figure 2:**
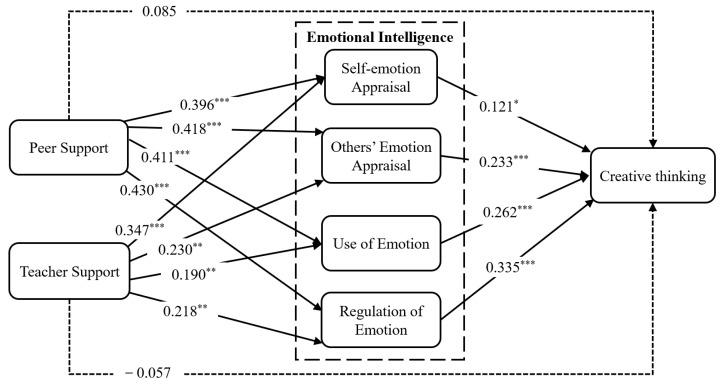
Hypothesized model with standardized path coefficients. Note: * *p* < 0.5; ** *p* < 0.01; *** *p* < 0.001.

As shown in Table 5 and Figure 2, peer and teacher support were positively and significantly linked with each dimension of emotional intelligence: self-emotion appraisal (β = 0.396, f^2^ = 0.158, *p* < 0.001; β = 0.347, f^2^ = 0.121, *p* < 0.001), others’ emotion appraisal (β = 0.418, f^2^ = 0.147, *p* < 0.001; β = 0.230, f^2^ = 0.044, *p* < 0.01), the use of emotion (β = 0.411, f^2^ = 0.132, *p* < 0.001; β = 0.190, f^2^ = 0.028, *p* < 0.01), and the regulation of emotion (β = 0.430, f^2^ = 0.156, *p* < 0.001; β = 0.218, f^2^ = 0.040, *p* < 0.01). However, the direct impacts of both peer and teacher support (β = 0.084, f^2^ = 0.010, *p* = 0.079; β = −0.057, f^2^ = 0.005, *p* = 0.168) on creative thinking were not observed statistically. Therefore, Hypotheses 1 and 2 were partially supported. Each dimension of emotional intelligence, such as self-emotion appraisal (β = 0.121, f^2^ = 0.021, *p* < 0.05), others’ emotion appraisal (β = 0.233, f^2^ = 0.084, *p* < 0.001), the use of emotion (β = 0.262, f^2^ = 0.108, *p* < 0.001), and the regulation of emotion (β = 0.335, f^2^ = 0.152, *p* < 0.001) was positively linked with creative thinking. Therefore, Hypothesis 3 was supported.

### 4.3. Testing for the Mediating Effect of Emotional Intelligence

According to Table 5, the direct influence of peer and teacher support on creative thinking was insignificant, while their effect could be extended through emotional intelligence. Therefore, the mediating effect of emotional intelligence between peer and teacher support and creative thinking was further examined. As is shown in Table 6, the direct impact of peer and teacher support on creative thinking were 0.084 (*p* > 0.05, [−0.011, 0.178]) and −0.057 (*p* > 0.05, [−0.137, 0.024]), respectively. The total effects of peer and teacher support on creative thinking were 0.481 (*p* < 0.001, [0.379, 0.582]) and 0.162 (*p* < 0.01, [0.051, 0.272]), respectively. The indirect effects of peer support on creative thinking with mediating roles of self-emotion appraisal, others’ emotion appraisal, the use of emotion, and the regulation of emotion were 0.048 (*p* < 0.05, [0.007, 0.088]), 0.097 (*p* < 0.001, [0.046, 0.149]), 0.108 (*p* < 0.001, [0.053, 0.162]), and 0.144 (*p* < 0.001, [0.079, 0.209]), respectively. Therefore, Hypotheses 4 and 5 were supported. Among these indirect paths between peer support and creative thinking, the regulation of emotion played the strongest mediating role, followed by the use of emotion, other’ emotion appraisal, and the weakest mediating effect displayed by self-emotion appraisal. In addition, the indirect effects of teacher support on creative thinking with mediating roles of self-emotion appraisal, others’ emotion appraisal, the use of emotion, and the regulation of emotion were 0.042 (*p* < 0.05, [0.005, 0.079]), 0.054 (*p* < 0.01, [0.018, 0.089]), 0.050 (*p* < 0.05, [0.008, 0.091]), and 0.073 (*p* < 0.01, [0.022, 0.124]), respectively. Among the indirect paths between teacher support and creative thinking, the regulation of emotion played the strongest mediating role, followed by others’ emotion appraisal, use of emotion, and self-emotion appraisal. It should be noted that this result may not fully align with the classical mediation criteria (e.g., Baron and Kenny 1986), yet does fit the definition of mediational models by others who suggests the statistical significance of indirect effects with a bootstrap approach as the core indicator of mediation models (e.g., Preacher and Hayes 2008).

### 4.4. Testing for the Moderating Effect of Emotion Regulation Strategies

**Figure 3:**
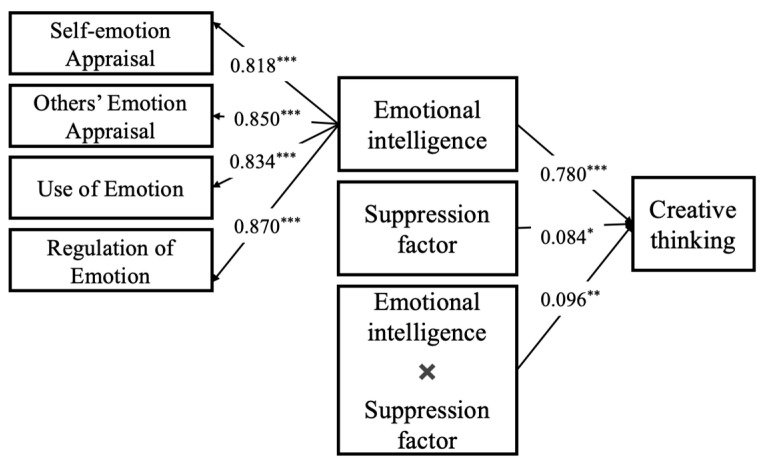
The influence of emotional intelligence on creative thinking: a moderating model of suppression factor, a kind of emotional regulation strategy. Note: * *p* < 0.5; ** *p* < 0.01; *** *p* < 0.001.

**Figure 4:**
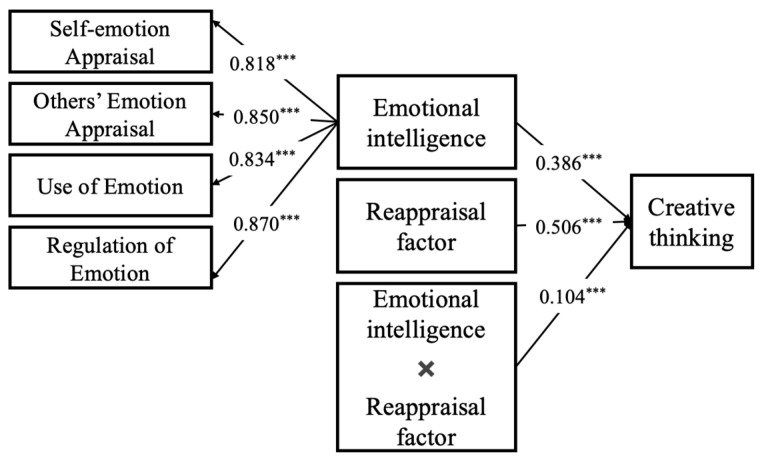
The influence of emotional intelligence on creative thinking: a moderating model of reappraisal factor, a kind of emotional regulation strategy. Note: *** *p* < 0.001.

In this study, creative thinking was used as the dependent variable, emotional intelligence as the independent variable, and emotion regulation strategies as moderating variables to build the moderating model (seeing Figure 3 and Figure 4). According to the results of the moderating analysis, the interaction of emotional intelligence and emotion regulation strategies (both reappraisal and suppression) had a significantly positive effect on creative thinking. That is, emotion regulation strategies of both reappraisal and suppression moderated the impact of emotional intelligence on creative thinking.

**Figure 5:**
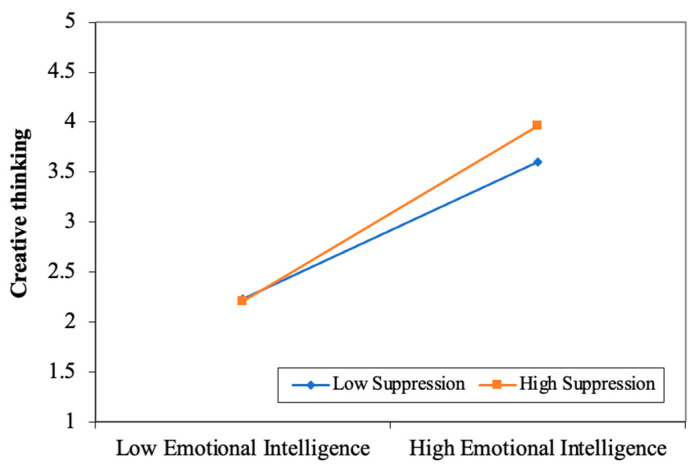
Interaction between emotional intelligence and suppression and its effect on creative thinking.

**Figure 6:**
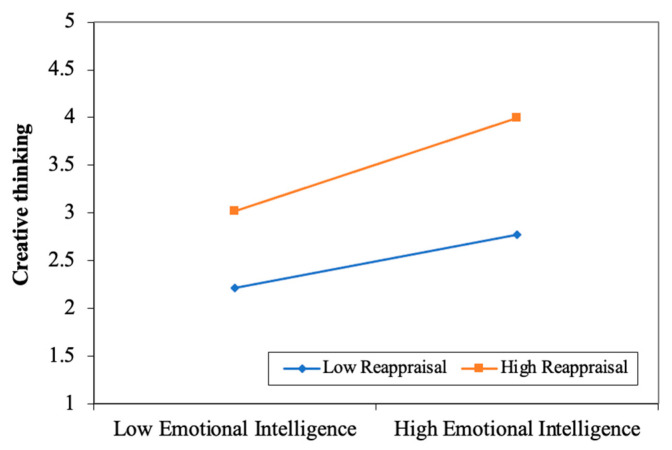
Interaction between emotional intelligence and reappraisal and its effect on creative thinking.

Furthermore, the simple plot analyses are given in Figure 5 and Figure 6 to interpret these moderating effects more vividly. The results showed that emotion regulation strategies of suppression and reappraisal could positively moderate the impact of emotional intelligence on creative thinking. Specifically, compared with low-level reappraisal or suppression, emotional intelligence offered a stronger positive influence on creative thinking when students were in high-level reappraisal or suppression. It implies that the positive relationship between emotional intelligence and creative thinking would be strengthened when reappraisal or suppression is higher.

## 5. Discussion

Cultivating students’ creative thinking receives increasing attention in contemporary society. This study aimed to explore the effect of perceived peer and teacher support on middle school students’ creative thinking with the mediating role of emotional intelligence and moderating role of emotion regulation strategies. The results revealed that the direct effects of peer and teacher support on creative thinking were not significant, while their effects would be extended to creative thinking with the mediating role of emotional intelligence. Additionally, the impact of emotional intelligence on creative thinking could be positively moderated by both reappraisal and suppression.

Specifically, peer support had a significant effect on four dimensions of emotional intelligence, including self-emotion appraisal, others’ emotion appraisal, the use of emotion, and the regulation of emotion. That is, students who perceived a higher level of peer support would exhibit stronger emotional intelligence. Conversely, students with poor peer relationships would show low emotional intelligence. This result was partially in concurrence with Hogan et al.’s (2010) research, claiming that peer support was a significant predictor of emotional intelligence for male college students in Canada. It also, to some extent, echoed Di Fabio and Kenny’s (2015) research, observing that three dimensions of emotional intelligence (e.g., intrapersonal, interpersonal, and stress management) were positively associated with perceived peer support among Italian youth. Therefore, the results suggest that educators should focus on creating a friendly classroom climate to increase social interaction and develop favorable peer relationships at schools and classes.

This study also found that teacher support was significantly associated with students’ emotional intelligence. That is, students who perceived more support from teachers would exhibit stronger emotional intelligence. This result was in line with Romano et al.’s (2020) research, suggesting that perceived emotional support from teachers was positively correlated with students’ emotional intelligence and negatively associated with academic anxiety and school burnout among Italian high school students. It means that not only does the emotional support students receive contribute to their emotional intelligence, such as self-control and well-being, but it also reduces their negative emotions (e.g., academic anxiety and school burnout). This finding also aligned with Atoum and Al-Shoboul’s (2018) research, suggesting that students’ perceived emotional support from teacher and peer was significantly and positively linked to their emotional intelligence including emotional awareness, management, and use. Therefore, this finding suggests that particular attention should be given to teacher support from psychological and academic aspects because it could develop students’ emotional intelligence.

Furthermore, this study also observed that the degree to which peer and teacher support affected the four dimensions of emotional intelligence was different. It was worth noting that among the direct paths from peer support to four subdomains of emotional intelligence, peer support offered the greatest impact on the regulation of emotion, while self-emotion appraisal had the smallest impact. Unlike peer support, self-emotion appraisal was most influenced by teacher support, while the least impact was observed in the use of emotion. Therefore, this finding suggests that in order to cultivate students’ emotional regulation ability, more attention should be given to enhancing their peer social interactions. Also, in order to strengthen students’ self-emotion appraisal, teachers should provide more opportunities to help students understand and exhibit their emotions.

This study also found that emotional intelligence had a positive impact on creative thinking. That is, individuals with a higher level of emotional intelligence exhibited stronger creative thinking abilities. This result was in concurrence with Murphy and Janeke (2009), suggesting that students with high emotional intelligence would commonly present complex creative thinking styles. Furthermore, this study extended previous findings regarding the relationship between emotional intelligence and creative thinking. Specifically, the different dimensions of emotional intelligence bore different influences on creative thinking. The regulation of emotion delivered the greatest influence on creative thinking, followed by the use of emotion, others’ emotion appraisal, and self-emotion appraisal. Therefore, this finding suggests that it is essential for educators to pay more attention to students’ emotional intelligence, especially the regulation of emotion, to cultivate and advance their creative thinking.

Moreover, the presented study observed that emotional intelligence mediated the relationship between peer and teacher support and creative thinking. First, according to the results of mediating analysis, peer support had a stronger indirect effect on creative thinking through emotional intelligence than that of teacher support. Second, the four dimensions of emotional intelligence bore different mediating effects. Specifically, the regulation of emotion had the strongest mediating effect between peer and teacher support and creative thinking, while self-emotion appraisal had the smallest effect. In addition, emotion regulation strategies moderated the effect of emotional intelligence on creative thinking. Specifically, both reappraisal and suppression bore positive moderating effects on the effect of emotional intelligence on creative thinking. When students were at a higher level of reappraisal or suppression, emotional intelligence would yield a stronger positive impact on creative thinking. One possible explanation is that using emotion regulation may lead to higher positive emotions, thereby enhancing the relationship between emotional intelligence and creative thinking. For instance, Song et al. (2019) demonstrated that reappraisal could effectively increase positive emotions and decrease negative emotions. Gross and Cassidy (2019) considered that emotion regulation of suppression may lead to positive emotions in some conditions. Importantly, Parke et al. (2015) interpreted how emotional intelligence shaped creative thinking using multi-methods and found that emotional intelligence could enable individuals to maintain and leverage positive emotion, thereby enhancing creative thinking. Especially, reappraisal, which occurred early, was more effective than suppression in regulating emotional effects. This result, to some extent, supported Gross’s (2002) research that an antecedent-focused form of emotion regulation is more effective than response modulation. Therefore, this study suggests that cultivating emotional intelligence, especially for the regulation of emotion, should be considered paramount. Moreover, students should also be encouraged to adopt emotional management strategies (both reappraisal of suppression) to promote their learning and thinking development.

## 6. Implications

In summary, these findings yield some implications for practitioners in the field of education to cultivate students’ creative thinking. First, both peer and teacher support were significantly associated with emotional intelligence. Therefore, more peer social interaction and teacher emotional support should be considered in teaching and learning processes for developing the students’ emotional intelligence. For instance, developing emotional intelligence training programs, such as helping students to perceive emotions in stories, music, or other activities, and facilitating students to use and feel emotion to convey feelings (Di Fabio and Kenny 2011). Second, students’ emotion regulation intelligence mediated the relationship between peer and teacher support and their creative thinking. Therefore, students’ creative thinking could be developed and cultivated if their competence in perceiving, appraising, and understanding emotions from others could be improved. Therefore, more attention from teachers and peer social interaction activities should be incorporated into the classroom to inspire and promote students’ creative thinking. Specifically, teachers should offer more opportunities to encourage students to understand and evaluate their emotions, such as designing educational games, like role playing and simulation, to cultivate students’ ability of assessing, using, and managing emotions (Napolitano et al. 2024). Peer social interaction activities, such as designing a simple self-expression game, should be encouraged to assist students in regulating their self-emotion. Last, this study found that both reappraisal and suppression can moderate the relationship between emotional intelligence and creative thinking. Therefore, students should be encouraged to learn how to manage and adjust their emotions and express themselves. Teachers should offer students more emotion regulation strategies in class to advance students’ ability to understand and regulate their emotions.

## 7. Limitations, Future Research, and Conclusions

In this study, several limitations should be acknowledged. First, the participants in this study were from a middle school in China. Examining whether the current findings would be valid for other districts and schools is essential. Second, the sample size in this study was not sufficiently large. Third, the data used in this study came from questionnaires, and only middle students were sampled. Therefore, the diversity of samples and the forms of surveys (i.e., interviews) should be increased in future work.

Despite the limitations, this study yields some interesting conclusions. Specifically, the present study provides a theoretical model to understand the internal mechanism regarding how to improve students’ creative thinking through peer and teacher support. The results demonstrate that emotional intelligence mediates the impact of peer and teacher support on students’ creative thinking. In addition, the moderating effect of emotion regulation strategies on the association between emotional intelligence and creative thinking is observed. Interestingly, emotion reappraisal exhibits a stronger moderating effect in this process than emotion suppression. Therefore, to advance students’ creative thinking, social support from peers and teachers, emotional intelligence, especially for the regulation of emotion, and emotion regulation strategies should be considered by practitioners and researchers.

## Figures and Tables

**Table 1:** The reliability and convergence validity of the measurement model in the first stage.

Constructs		Significance Test of Parameters				Composite Reliability	Convergence Validity
		Estimate	STDEV	*t*	*p*	CR	AVE
COE	COE1	0.885	0.012	75.466	***	0.904	0.704
	COE2	0.898	0.012	74.076	***		
	COE3	0.885	0.014	65.439	***		
	COE4	0.666	0.043	15.415	***		
INE	INE1	0.846	0.022	39.194	***	0.885	0.720
	INE2	0.881	0.014	64.801	***		
	INE3	0.817	0.031	26.545	***		
DOT	DOT1	0.891	0.017	53.850	***	0.892	0.805
	DOT2	0.903	0.012	76.005	***		
FLY	FLY1	0.868	0.018	48.512	***	0.872	0.696
	FLY2	0.750	0.038	19.754	***		
	FLY3	0.878	0.016	55.837	***		
INS	INS1	0.865	0.018	48.768	***	0.954	0.725
	INS2	0.832	0.024	34.756	***		
	INS3	0.894	0.014	63.083	***		
	INS4	0.676	0.041	16.372	***		
	INS5	0.872	0.018	48.493	***		
	INS6	0.868	0.016	53.322	***		
	INS7	0.913	0.012	77.109	***		
	INS8	0.868	0.018	47.371	***		
SED	SED1	0.829	0.024	33.870	***	0.937	0.750
	SED2	0.842	0.024	35.715	***		
	SED3	0.894	0.016	56.923	***		
	SED4	0.885	0.016	55.015	***		
	SED5	0.877	0.017	52.163	***		

Note: *** *p* < 0.001. COE = courage; INE = inquisitiveness; DOT = doubt; FLY = flexibility; INS = innovation search; SED = self-discipline; STDEV = standard deviation; AVE = average variance extracted.

**Table 2:** The discriminant validity of the measurement model in the first stage.

Constructs	Correlations of the Latent Variables					
	COE	INE	DOT	FLY	INS	SED
COE	0.839					
INE	0.725	0.848				
DOT	0.668	0.748	0.897			
FLY	0.651	0.648	0.720	0.834		
INS	0.757	0.783	0.725	0.741	0.851	
SED	0.700	0.726	0.679	0.742	0.869	0.866

Note: Values of diagonal represent the square root of AVE.

**Table 3:** The reliability and convergence validity of the measurement model in the second stage.

Constructs		Significance Test of Parameters				Composite Reliability	Convergence Validity
		Estimate	STDEV	*t*	*p*	CR	AVE
CRT	COE	0.850	0.018	48.239	***	0.953	0.772
	INE	0.874	0.023	38.347	***		
	DOT	0.852	0.018	48.370	***		
	FLY	0.855	0.018	47.026	***		
	INS	0.932	0.008	119.040	***		
	SED	0.904	0.010	93.257	***		
OEA	OEA1	0.824	0.022	37.499	***	0.911	0.719
	OEA2	0.882	0.014	64.887	***		
	OEA3	0.827	0.027	30.749	***		
	OEA4	0.856	0.018	46.542	***		
PES	PES1	0.895	0.016	55.413	***	0.932	0.821
	PES2	0.931	0.011	87.942	***		
	PES3	0.893	0.016	56.469	***		
ROE	ROE1	0.921	0.013	69.927	***	0.956	0.878
	ROE2	0.930	0.011	85.939	***		
	ROE3	0.959	0.005	187.878	***		
SEA	SEA1	0.866	0.023	38.410	***	0.933	0.822
	SEA2	0.935	0.010	94.840	***		
	SEA3	0.918	0.012	74.491	***		
TES	TES1	0.812	0.027	30.467	***	0.910	0.716
	TES2	0.881	0.015	58.464	***		
	TES3	0.802	0.024	33.206	***		
	TES4	0.886	0.015	58.351	***		
UOE	UOE1	0.920	0.011	82.070	***	0.949	0.861
	UOE2	0.948	0.007	130.130	***		
	UOE3	0.916	0.015	61.159	***		

Note: *** *p* < 0.001. CRT = creative thinking; OEA = others’ emotion appraisal; PES = peer support; ROE = regulation of emotion; SEA = self-emotion appraisal; TES = teacher support; UOE = use of emotion; STDEV = standard deviation; AVE = average variance extracted.

**Table 4:** The discriminant validity of the measurement model in the second stage.

Constructs	Correlations of the Latent Variables						
	CRT	OEA	PES	ROE	SEA	TES	UOE
CRT	0.878						
OEA	0.691	0.848					
PES	0.59	0.574	0.906				
ROE	0.755	0.637	0.578	0.937			
SEA	0.635	0.641	0.632	0.594	0.907		
TES	0.488	0.513	0.678	0.51	0.616	0.846	
UOE	0.712	0.566	0.54	0.69	0.566	0.469	0.928

Note: values of diagonal represent the square root of AVE.

**Table 5:** Assessment of the structural model.

Hypothesis	Paths	Significance Test of Hypothesis				95% CI		Conclusion	Model Fit Index		
		Std Beta	STDEV	*t*	*p*	2.50%	97.50%		R^2^	f^2^	Q^2^
H1e	PES -> SEA	0.396	0.070	5.675	***	0.259	0.533	Supported	0.464	0.158	0.376
H3b	TES -> SEA	0.347	0.065	5.316	***	0.219	0.475	Supported		0.121	
H2a	PES -> UOE	0.411	0.066	6.183	***	0.281	0.541	Supported	0.311	0.132	0.264
H3c	TES -> UOE	0.190	0.066	2.860	**	0.060	0.320	Supported		0.028	
H1d	PES -> ROE	0.430	0.066	6.523	***	0.301	0.559	Supported	0.359	0.156	0.312
H3a	TES -> ROE	0.218	0.069	3.156	**	0.083	0.353	Supported		0.040	
H1c	PES -> OEA	0.418	0.067	6.230	***	0.286	0.549	Supported	0.357	0.147	0.248
H2e	TES -> OEA	0.230	0.068	3.385	**	0.097	0.363	Supported		0.044	
H1b	PES -> CRT	0.084	0.048	1.759	0.079	−0.010	0.177	Not	0.699	0.010	0.529
H2d	TES -> CRT	−0.057	0.041	1.377	0.168	−0.138	0.024	Not		0.005	
H2c	SEA -> CRT	0.121	0.047	2.573	*	0.029	0.213	Supported		0.021	
H1a	OEA -> CRT	0.233	0.045	5.160	***	0.145	0.322	Supported		0.084	
H3d	UOE -> CRT	0.262	0.056	4.716	***	0.153	0.371	Supported		0.108	
H2b	ROE -> CRT	0.335	0.053	6.334	***	0.231	0.438	Supported		0.152	

Note: * *p* < 0.5; ** *p* < 0.01; *** *p* < 0.001; Std Beta = standard path coefficients; STDEV = standard deviation; 95% CI = 95% confidence intervals.

**Table 6:** Mediating effect analysis.

Paths	Significance Test of Hypothesis				95% CI	
	Std Beta	STDEV	*t*	*p*	2.50%	97.50%
**Total Effect**						
PES -> CRT	0.481	0.052	9.284	0.000	0.379	0.582
TES -> CRT	0.162	0.056	2.872	0.004	0.051	0.272
**Indirect Effect**						
TES -> ROE -> CRT	0.073	0.026	2.817	0.005	0.022	0.124
PES -> OEA -> CRT	0.097	0.026	3.727	0.000	0.046	0.149
PES -> SEA -> CRT	0.048	0.021	2.317	0.021	0.007	0.088
TES -> UOE -> CRT	0.050	0.021	2.351	0.019	0.008	0.091
PES -> ROE -> CRT	0.144	0.033	4.320	0.000	0.079	0.209
PES -> UOE -> CRT	0.108	0.028	3.885	0.000	0.053	0.162
TES -> OEA -> CRT	0.054	0.018	2.973	0.003	0.018	0.089
TES -> SEA -> CRT	0.042	0.019	2.235	0.025	0.005	0.079
**Direct Effect**						
PES -> CRT	0.084	0.048	1.734	0.083	−0.011	0.178
TES -> CRT	−0.057	0.041	1.385	0.166	−0.137	0.024

Note: Std Beta = standard path coefficients; STDEV = standard deviation; 95% CI = 95% confidence interval.

## Data Availability

The data generated and analyzed in this study are available from the corresponding author on reasonable request.
